# Assessment of the prophylactic speed of kill of Frontline Tri-Act^®^ against ticks (*Ixodes ricinus* and *Rhipicephalus sanguineus*) on dogs

**DOI:** 10.1051/parasite/2016002

**Published:** 2016-01-21

**Authors:** Frédéric Beugnet, Lénaïg Halos, Julian Liebenberg, Josephus Fourie

**Affiliations:** 1 Merial SAS 29 Avenue Tony Garnier 69007 Lyon France; 2 ClinVet International (Pty) Ltd PO Box 11186 9321 Universitas South Africa

**Keywords:** Ticks, *Ixodes ricinus*, *Rhipicephalus sanguineus*, Permethrin, Fipronil, Dog, Efficacy, Speed of kill, Frontline Tri-Act^®^/Frontect^®^

## Abstract

The objective of the study was to assess the speed of kill of a single topical treatment with a combination of fipronil and permethrin (Frontline Tri-Act^®^/Frontect^®^) against experimental infestations of *Ixodes ricinus* and *Rhipicephalus sanguineus* ticks on dogs. In this parallel group designed, randomised, single centre, controlled efficacy study, 16 healthy adult dogs were allocated to two groups: 8 dogs were treated with the topical combination on Day 0 and the other 8 dogs served as untreated controls. Each dog was exposed in a crate to 100 *I. ricinus* (50 females, 50 males) and 50 *R. sanguineus* (25 males, 25 females) on Days 2, 7, 14, 21 and 28. Ticks were counted *in situ* at 6 and 12 h after exposure and removed at 24 h after exposure. Frontline Tri-Act^®^ was effective (≥90%) against both *R. sanguineus* and *I. ricinus* tick infestations at 6, 12 and 24 h after exposure, from 2 to 28 days after treatment. This is the first time that a topical ectoparasiticide has demonstrated a preventive killing effect against these two tick species in 6 h for a full month.

## Abstract (fr)

L’objectif de cette étude était d’évaluer la vitesse d’action d’une application topique unique d’une combinaison de fipronil et de perméthrine (Frontline Tri-Act^®^/Frontect^®^) lors d’infestations expérimentales de chiens par les tiques *Ixodes ricinus* et *Rhipicephalus sanguineus*. Dans cette étude contrôlée, conduite en parallèle de façon randomisée, 16 chiens adultes sains ont été divisés en deux groupes : 8 chiens traités avec la combinaison au jour 0 et 8 chiens servant de contrôles non traités. Chaque chien a été exposé dans un boxe à 100 tiques *I. ricinus* (50 femelles, 50 mâles) et 50 tiques *R. sanguineus* (25 femelles, 25 mâles) aux jours 2, 7, 14, 21 et 28. Les tiques étaient comptées *in-situ* à 6 et 12 heures après chaque exposition et retirés 24 heures après exposition. Frontline Tri-Act^®^ a été actif (≥90%) vis-à-vis des deux espèces de tiques, *R. sanguineus* et *I. ricinus,* à 6, 12 et 24 heures après exposition, du jour 2 au jour 28 après le traitement. C’est la première fois qu’un acaricide topique démontre une activité préventive vis-à-vis d’une infestation par ces deux espèces de tiques en 6 heures durant un mois.

## Introduction

Ticks are among the most common external parasites of dogs [4, 5]. They have the potential to transmit pathogenic agents to both dogs and their owners [2, 4]. *Ixodes ricinus* is the main vector of Lyme borreliosis in Europe. *I. ricinus* is expanding in range and period of activity during the year, possibly as a result of climate change [3, 6, 14]. *Rhipicephalus sanguineus* has a worldwide distribution and is also a common tick species in the Mediterranean region. It can transmit a variety of pathogens to dogs, including *Babesia canis vogeli, Ehrlichia canis, Anaplasma platys* and *Hepatozoon canis*. It is also capable of transmitting pathogens to humans such as *Rickettsia conorii*, the agent of Mediterranean spotted fever [2, 4]. Preventing disease transmission is an important concern to veterinarians and dog owners, especially in the context of the expansion of several tick-borne diseases confirmed in Europe [5, 7, 23, 27].

Fipronil has been used as a topical spot on for dogs and cats to kill fleas and ticks since the mid-1990s. Combinations of fipronil and various other anti-parasitic compounds are also available. Pyrethroid compounds are known for their ability to repel ticks and flying insects, as well as induce rapid neurotoxicity (known as the “knock-down” effect) in arthropods [1]. Therefore, a combination of fipronil and the pyrethroid permethrin can provide both repellency and increased acaricidal efficacy against ticks. Recently, a combination of fipronil and permethrin (Frontline Tri-Act^®^/Frontect^®^) was tested for its repellent and acaricidal efficacy against *I. ricinus*, *Dermacentor reticulatus* and *R. sanguineus* ticks [9–11]. These efficacy studies demonstrated high efficacy against ticks over a full month [9], but the assessments were conducted at 48 h after treatment or infestation following classical efficacy guidelines in order to obtain registration [12, 20, 25] and the speed of kill within the first 24 h of exposure was not evaluated.

Pathogen transmission depends on the duration of attachment required by ticks to transmit specific pathogens such as *B. canis* and *E. canis* [19]. In general, *Babesia* protozoans require several days (36–72 h) for their sporoblasts to mature into infective sporozoites within the tick’s salivary glands before they can be transmitted. Bacterial pathogens, such as *Anaplasma phagocytophilum,* require 24–36 h to be transmitted by nymphal *Ixodes scapularis* ticks [8]. In a recent study, the attachment duration needed for infected *R. sanguineus* ticks before they could transmit *E. canis* was determined *in vivo* as well as *in vitro* [18, 24]. The study revealed that transmission of *E. canis* starts within a few hours (3 h on dogs and 8 h on artificial membranes), an interval considerably shorter than presumed previously. These findings highlight the need for further research concerning the actual speed of transmission of tick-borne pathogens.

To reduce the risk of pathogen transmission by acaricidal treatment, two characteristics that can be valuable include repellent activity, and demonstrated rapid speed of kill [1, 20]. Following WAAVP and EMA guidelines, repellency is calculated by comparing the number of ticks on treated animals versus controls at 24 h or before (usually 4 h) [12, 25]. Several experimental studies have demonstrated that acaricidal treatments based on fipronil and/or pyrethroids can reduce the transmission of pathogens by infected ticks to dogs [15, 16, 17, 22, 28]. Recently, the combination of fipronil and permethrin in a similar spot on formulation demonstrated preventive activity against the transmission of *Babesia canis* [26]. In addition, significant reduction of the risk of transmission of *B. canis* and *E. canis* was also demonstrated for the Frontline Tri-Act formulation [21]. To explain this preventive activity against tick-borne disease transmission, the repellent effect of the combination of fipronil and permethrin (Frontline Tri-Act^®^) against *I. ricinus* and *R. sanguineus* was studied following standardised guidelines, and published recently [11]. Knowing that under real conditions, repellency cannot be considered as complete, the speed of kill must also be considered. Current regulatory guidelines indicate that claiming efficacy against ticks requires >90% efficacy within 48 h [12]. Based on the latest knowledge on the dynamics of pathogen transmission and in areas at high risk for vector-borne disease transmission, this may be too long in some instances. Thus, the objective of the present study was to assess the speed of kill of the combination of fipronil and permethrin (Frontline Tri-Act^®^) against new infesting *I. ricinus* and *R. sanguineus* ticks at 6, 12 and 24 h after tick exposure.

## Materials and methods

This study was an experimental controlled study in dogs conducted in accordance with Good Clinical Practices (GCP) as described in International Cooperation on Harmonisation of Technical Requirements for Registration of Veterinary Medicinal Products (VICH) (Table 1) [13]. It was a parallel group, randomised, blinded, controlled efficacy study, conducted following the standard methods for evaluating the efficacy of parasiticides for the treatment, prevention and control of tick infestations [12, 25].

**Table 1.** Summary of the study schedule.

Acclimatisation	Tick infestations^1^	Ranking and allocation to groups	Treatment administration
Days −7 to −1	Days −6 (*R. sanguineus* only), 2, 7, 14, 21 and 28	Day −3	Day 0
Assessments 6 h (±15 min) *in situ* tick palpation counts	Assessments 12 h (±15 min) *in situ* tick palpation counts	Assessments 24 h (±30 min) tick counts and removal	
Days 2, 7, 14, 21 and 28	Days 2, 7, 14, 21 and 28	Days −5 (*R. sanguineus* only), 3, 8, 15, 22 and 29	

1 Dogs were sedated and confined to infestation crates for 4 h following initial exposure to ticks.

### Animals

Sixteen healthy mixed breed dogs, weighing 9.6 kg to 24.2 kg, were studied. None had been exposed to ectoparasiticides for at least 12 weeks prior to the start of the study. On Day −5, the dogs were randomly allocated to the treatment and control groups based on pre-treatment live attached tick (*R. sanguineus*) counts performed at 24 h after exposure.

The dog cages were part of an indoor animal unit, environmentally controlled for temperature (~20 °C ± 4 °C). A photoperiod of 12 h light: 12 h darkness was maintained. The floor size of each dog cage was approximately 2.0 m × 3.0 m. The animals were kept individually in cages and no contact between dogs was possible during the study. Animals were handled in compliance with the Merial Ethical Committee standards and in compliance with the South African National Standard “SANS 10386:2008 The care and use of animals for scientific purposes” [29]. The dogs were observed for general health conditions daily throughout the study. In addition, all dogs were observed hourly for 4 h following administration of the treatment on Day 0.

### Allocation and treatment

Dogs were ranked by pre-treatment live attached *R. sanguineus* tick counts within sex. They were then randomly allocated to one of the two groups. Animals in Group 1 (*n* = 8) served as untreated control dogs. Dogs in Group 2 (*n* = 8) were treated topically on Day 0 with Frontline Tri-Act^®^4 (fipronil 6.76% w/v and permethrin 50.48% w/v) according to label instructions (i.e. 2 mL for dogs weighing 10 to <20 kg and 4 mL for dogs weighing 20 to <40 kg). The treated dogs weighed 10.4–24.2 kg. The administered doses ranged between 7 mg fipronil + 52.4 mg permethrin, administered to a 19.2 kg dog, to 12.9 mg fipronil + 96.9 mg permethrin, administered to a 10.4 kg dog.

### Tick infestations and counts

Laboratory-bred strains of *I. ricinus* and *R. sanguineus* of European origin (originally collected in France) were used for the tick exposures. The ticks were adults, unfed and at least one week old. They tested negative by PCR for several tick-borne pathogens (i.e. genus-specific qualitative PCRs for *Anaplasma*, *Borrelia*, *Ehrlichia* and *Babesia*). Each dog was exposed to 50 *I. ricinus* females (with an additional 50 male *I. ricinus* ticks to stimulate female attachment) and 50 *R. sanguineus* (25 males, 25 females) on Days 2, 7, 14, 21 and 28. The dogs were sedated prior to exposure and confined to crates for up to 4 h following tick challenge. Ticks were deposited on the ground of the crate on both sides of the dogs, at 10–20 cm away from the abdomen or the dorsal line of each dog.

The time at which each animal was exposed to ticks was recorded. This was done to ensure that counting and removal of ticks was as close as possible to the specified target times (6 h ± 15 min and 12 h ± 15 min after the start of exposure for *in situ* counts and 24 h ± 30 min after the start of exposure for tick counts and removal). Ticks were found by direct observation following parting of the hair coat and palpation.

At 24 h after the start of tick exposure in the crates, all dogs were combed to ensure that all ticks were counted and removed. Areas examined, not necessarily in this order, were: outside hind legs, including feet, tail and anal areas, lateral areas, not including shoulders, abdominal area, from chest to inside hind legs, fore legs and shoulders, including feet, all neck and head areas, and dorsal strip from shoulder blades to base of tail.

Following the WAAVP guideline, ticks were recorded as live or dead and free or attached [25].

### Data analysis

The primary criterion was the assessment of the acaricidal effect. It was calculated based on the number of live ticks on dogs at 6 h, 12 h and 24 h.

Calculations were based on arithmetic means. Concerning *I. ricinus*, only females were recorded and included in the calculations as males do not attach or take a blood meal, while both females and males were taken into account for *Rhipicephalus*.

The percentage efficacy was calculated as follows:

$$\mathrm{Efficacy}\;(\%)\;\mathrm{against}\;\mathrm{ticks}\;=\;100\times (M_{c}\;–M_{t})\;/M_{c},$$

where:

*M*_c_ = Arithmetic mean number of live ticks (free or attached) on dogs in the negative control group (group 1) at a specific time point.
*M*_t_ = Arithmetic mean number of live ticks (free or attached) on dogs in the Frontline Tri-Act^®^ groups (group 2) at a specific time point.

The Committee for Medicinal Products for Veterinary Use (CVMP) guideline “Guideline for the testing and evaluation of the efficacy of anti-parasitic substances for the treatment and prevention of tick and flea infestations in cats and dogs” states that at least six animals should be used per group [12]. Eight animals per group were used in this study, which was in compliance with the guidelines.

The statistical unit was the specific group if central values of a group were compared. In other cases, the statistical unit was the individual animal.

The groups were compared at each time point by a one-way Analysis of Variance (ANOVA) with an administration effect on the untransformed tick count data. SAS Version 9.3 TS Level 1M2 was used for all the statistical analyses. The level of significance of the formal tests was set at 5%, all tests were two-sided.

## Results

No adverse health effects related to the treatment occurred during the study.

The arithmetic mean tick counts and efficacies are summarised in Tables 2 and 3 for *R. sanguineus* and *I. ricinus*, respectively. The arithmetic mean tick count recorded in the untreated control group 1 ranged from 15.6 to 21.8 for *R. sanguineus* and from 7.0 to 12.3 for *I. ricinus* at 24 h after exposure, indicating an adequate tick infestation throughout the study. Statistically significantly (*p* < 0.05) fewer ticks were recorded for the treated group compared to the untreated control group at all assessment time points and days for both tick species.

**Table 2.** Efficacy against *Rhipicephalus sanguineus* (arithmetic means).

Day	Time point (h)	Tick mean (Eff.%)		*p*-value
Group		Group 1	Group 2	
Day 2	+6	27.5	2.5 (90.9)	<.0001
Day 7	+6	27.0	0.1 (99.5)	<.0001
Day 14	+6	21.9	0.5 (97.7)	<.0001
Day 21	+6	26.4	1.6 (93.8)	<.0001
Day 28	+6	22.8	1.1 (95.1)	<.0001
Day 2	+12	24.4	0.6 (97.4)	<.0001
Day 7	+12	23.0	0 (100)	<.0001
Day 14	+12	19.8	0 (100)	<.0001
Day 21	+12	22.8	0.8 (96.7)	<.0001
Day 28	+12	22.0	0.5 (97.7)	<.0001
Day 3	+24	15.6	0 (100)	<.0001
Day 8	+24	20.4	0 (100)	<.0001
Day 15	+24	17.3	0 (100)	<.0001
Day 22	+24	21.8	0.1 (99.4)	<.0001
Day 29	+24	21.5	0.5 (97.7)	<.0001

**Table 3.** Efficacy against *Ixodes ricinus* (arithmetic means).

Day	Time point (h)	Tick mean (Eff.%)		*p*-value
Group		Group 1	Group 2	
Day 2	+6	10.1	0.3 (97.5)	<.0001
Day 7	+6	15.9	0 (100)	<.0001
Day 14	+6	11.4	0 (100)	<.0001
Day 21	+6	9.5	0.6 (93.4)	0.0001
Day 28	+6	9.6	0 (100)	<.0001
Day 2	+12	9.5	0.1 (98.7)	<.0001
Day 7	+12	15.5	0 (100)	<.0001
Day 14	+12	10.3	0.1 (98.8)	<.0001
Day 21	+12	7.4	0.3 (96.6)	0.0004
Day 28	+12	8.9	0 (100)	<.0001
Day 3	+24	9.5	0 (100)	<.0001
Day 8	+24	12.3	0 (100)	<.0001
Day 15	+24	7.8	0 (100)	0.0001
Day 22	+24	7.0	0.1 (98.2)	0.0003
Day 29	+24	8.6	0 (100)	0.0002

Frontline Tri-Act^®^ was effective (>90.9% and >93.4%) against *R. sanguineus* and *I. ricinus* tick infestations, respectively, based on arithmetic mean tick counts, from as soon as 6 h after exposure from Days 2 to 28. Efficacy was >96.7% and >96.6% against *R. sanguineus* and *I. ricinus*, respectively, for the 28-day period if we consider tick counts at 12 h.

## Discussion

This study demonstrated that a combination of fipronil and permethrin has high acaricidal effect against two of the most common tick species in Europe, *I. ricinus* and *R. sanguineus*. These results complement similar experiments using the same combination product against the same tick species, but with acaricidal efficacy calculated at 48 h [9, 10]. In these studies, 48 h efficacy against *R. sanguineus* (two experiments) ranged from 94.4% to 100% during the month, and 48 h efficacy against *I. ricinus* ranged from 99.2% to 100%. It also complements the studies demonstrating the repellent activity against these tick species [11] as well as the capacity to prevent the transmission of either *Babesia canis* (94% prevention against infective challenges in a one-month experiment) and *Ehrlichia canis* (85% prevention against infective tick challenges in a two-month experiment) [21].

Based on the tick counts recorded throughout the study on the untreated dogs, this study met the guideline recommendations for adequate infestation in the control group for *Rhipicephalus* ticks at all time points (i.e. average of 12.5) [20, 25]. The infestation rate for *Ixodes* ticks in the control group was lower than expected. Nevertheless, the number of ticks found on the treated dogs at all time points (means of 0 to 0.6 for *I. ricinus* and 0.1 to 0.8 for *R. sanguineus*) was very limited and significantly lower than for the control counts.

At the 6 h time point, an effectiveness of at least 90% was calculated for a full month, which is a key fact in order to reduce the risk of transmission of tick-borne pathogens [19, 20]. Between 6 and 24 h, most of the ticks fall off as demonstrated by the efficacy percent observed at 24 h (100% and 99.4% for *Ixodes* and *Rhipicephalus*, respectively).

In a recently published paper, it was shown that the combination permethrin-fipronil product killed *I. ricinus* ticks within 4 h of exposure with efficacy >91.1% for the full month and killed *R. sanguineus* ticks at 4 h with acaricidal efficacy ≥94.7% after exposure from Day 2 to Day 21 and 71.4% on Day 28 [11]. In the present study, the efficacy remained >90.9% against *R. sanguineus* at 6 h count for a full month. This is the first time that a topical acaricidal product has been shown to provide such a rapid prophylactic speed of kill against both *I. ricinus* and *R. sanguineus*. The high killing effect observed during this study is most probably due to the combined effect of both permethrin and fipronil.

In addition to this quick and sustained acaricidal effect, the combination permethrin-fipronil product demonstrated sustained repellency against both *I. ricinus* and *R. sanguineus* ticks at 4 h and 24 h [11]. The addition of both repellency and acaricidal effect is important in order to reduce the risk of transmission of pathogens by ticks [19, 20]. It also reduces the possibility for dog owners to see attached and engorging ticks on their dogs.

## Conflict of interest

The work reported herein was funded by Merial Limited. All authors were employees or contractors of Merial.

## Disclaimer

Frontline Tri-Act^®^ (Frontect^®^) are registered trademarks of Merial in France and pending registration in other countries. All other marks are the property of their respective owners.

This document is provided for scientific purposes only. Any reference to a brand or trademark herein is for informational purposes only and is not intended for a commercial purpose or to dilute the rights of the respective owner(s) of the brand(s) or trademark(s).
